# Increase maize productivity and water use efficiency through application of potassium silicate under water stress

**DOI:** 10.1038/s41598-020-80656-9

**Published:** 2021-01-08

**Authors:** M. A. Gomaa, Essam E. Kandil, Atef A. M. Zen El-Dein, Mamdouh E. M. Abou-Donia, Hayssam M. Ali, Nader R. Abdelsalam

**Affiliations:** 1 Plant Production Department, Faculty of Agriculture, Saba Basha, Alexandria University, Alexandria, 21531 Egypt; 2 Agriculture Research Center (ARC), Etay El-Baroud Research Station, El-Beheira, Egypt; 3 Botany and Microbiology Department, College of Science, King Saud University, P.O. Box 2455, Riyadh, 11451 Saudi Arabia; 4 Timber Trees Research Department, Sabahia Horticulture Research Station, Horticulture Research Institute, Agriculture Research Center, Alexandria, 21526 Egypt; 5 Agricultural Botany Department, Faculty of Agriculture (Saba Basha), Alexandria University, Alexandria, 21531 Egypt

**Keywords:** Biological techniques, Ecology, Plant sciences

## Abstract

In Egypt, water shortage has become a key limiting factor for agriculture. Water-deficit stress causes different morphological, physiological, and biochemical impacts on plants. Two field experiments were carried out at Etay El-Baroud Station, El-Beheira Governorate, Agriculture Research Center (ARC), Egypt, to evaluate the effect of potassium silicate (K-silicate) of maize productivity and water use efficiency (WUE). A split-plot system in the four replications was used under three irrigation intervals during the 2017 and 2018 seasons. Whereas 10, 15, and 20 days irrigation intervals were allocated in main plots, while the three foliar application treatments of K-silicate (one spray at 40 days after sowing; two sprays at 40 and 60 days; and three sprays at 40, 60, and 80 days, and a control (water spray) were distributed in the subplots. All the treatments were distributed in 4 replicates. The results indicated that irrigation every 15 days gave the highest yield in both components and quality. The highly significant of (WUE) under irrigation every 20 days. Foliar spraying of K-silicate three times resulted in the highest yield. Even under water-deficit stress, irrigation every fifteen days combined with foliar application of K-silicate three times achieved the highest values of grain yield and its components. These results show that K-silicate treatment can increase WUE and produce high grain yield requiring less irrigation.

## Introduction

Maize (*Zea mays* L.) is considering the third highest important staple food crop by area and production in Egypt and the world. In Egypt, the cultivated area occupies approximately 935,778 ha, which produces up to seven million tons of grains with an average yield of 7.60 ton/ha^1^. Yellow maize cultivation in Egypt is used in the production of food products to reduce imports of meat and milk^2,3^.

In Egypt, the water resources are limited and restrict many crop production especially in newly reclaimed lands due to the establishment of intensive agricultural production in the Nile Delta and valley area. The agricultural area consumes more than 84% of the available water resources^4,5^. Three factors affect the agricultural water use such as the water needs (evapotranspiration) by the crop, water availability, and water holding capacity of the soil^6^. Also, climate changes, such as altered precipitation and temperature systems, have had negative effects on crop quantity and yields. Seasonal global temperatures have increased, with even larger changes observed in several regions^7^. About 30% of the variation in average global yields of wheat, rice, maize, soybeans, barley, and sorghum resulted from seasonal precipitation and temperature changes^8^. The uneven spatiotemporal distribution of water resources has led to water shortages that limit maize yield in arid and semiarid areas^9^.

Water stress is one of the most vital environmental stresses that generally results in decreased growth and final yield in many crops^10–12^. The vegetative growth stage is inhibited by water stress and results in decreased overall growth, leaf area, and yield in maize plants^13^. When the soil water content is suboptimal during the vegetative growth and grain-filling stages, high maize yield can be obtained through full irrigation at the flowering stage^14^. Maize exposed to light water stress throughout the early growth and late grain-filling stages exhibited some water stress tolerance due to the low water demand of these stages^15^. Maximizing crop productivity by utilizing accessible irrigation is a global sustainable goal and the center of much research because agriculture is the core consumer of freshwater^16,17^.

Silicon (Si) is an important nutrient for high-yielding crop species such as cereals, legumes, and vegetables. Si can be detected in limiting levels under some growing conditions, and without Si, plants may suffer subtle nutrient deficiency. Si deficiency decreased photosynthesis, lowered Brix, increased disease prevalence and insect attacks, increased wilting, and enhanced postharvest fall. All these symptoms are signs of stress^18,19^. In addition, Si aids in healthy plant development and is essential for cell development and differentiation^20^. Si treatment can alleviate drought stress, salt stress, heat stress, and oxidative damage. The beneficial role of Si in drought conditions is mainly due to enhanced water retention aiding photosynthesis^21–25^. Potassium silicate (K-silicate) is a source of highly soluble K and Si. K-silicate encloses no volatile organic compounds, and application does not result in the release of hazardous or environmentally persistent by-products^26^. K-silicate application to the soil of some cereal species under water-deficit irrigation produced the highest biomass yield responses across species. Si may work as a growth regulator and has the potential to increase plant growth under drought stress^27^. The growth and yield of maize were highly responsive to Si application under stressed conditions^5,28^. Spraying K-silicate and other nanomaterials has the potential to reduce the harmful effects of drought stress on crops^29–32^.

Silicon was revealed to be helpful to plants by reducing the absorption of high levels of nutrients, which may be present in soil, in plant tissue and by preventing tissue damage^33^. Si has a role in ethylene inhibition, which decreases the speed of aging and death of harvested plant parts. Si is beneficial for chlorophyll content and helps crops maintain freshness longer with better appearance. Thus, Si has a positive impact on both crop yield and quantity^34^. The grain yield of rice was improved with the application of Si fertilizer^35^**.** Also^12^, revealed that spraying of nanoparticles of SiO_2_, improved the growth and yield parameters of strawberry plants under water stress.

Here, we investigate irrigation intervals to achieve the highest productivity and water use efficiency (WUE). We also evaluate the performance of K-silicate foliar spray to mitigate water-deficit stress on maize yield and examine its effect on WUE.

## Materials and methods

### Experimental site

Two field experiments were conducted at the Experimental Farm of Etay El-Baroud Station, El-Beheira Governorate, Agriculture Research Center (ARC), Egypt, during the 2017 and 2018 growing seasons. Where, Etay El-Baroud is located at 30° 53′ 13.2″ N, 30° 39′ 54″ E ,El-Beheira Governorate, Egypt on the agricultural road between Cairo and Alexandria, north of Kafr El Zayat and south of Damanhour, and is 140 km away from Cairo and 84 km from Alexandria (Table 1). The experiments were carried out to study the effect of irrigation intervals, foliar application of Si and their interaction on maize productivity in 2017 and 2018 seasons.

**Table 1:** Meteorological of the experimental site in both seasons. *Source*: Central Laboratory for Agriculture Climate, the Agriculture Research Center, Egypt.

Month	HC air temperature (C0)				HC relative humidity (%)	
	Seasons					
	2017	2018	2017	2018		
	Minimum		Maximum		2017	2018
May	17.1	22.0	38.0	40.9	65.0	62.1
June	20.5	20.3	40.2	40.8	73.0	71.8
July	19.1	20.5	37.3	35.9	74.8	79.0
August	17.4	20.0	36.7	35.4	72.2	77.3
September	17.0	19.0	35.7	32.4	68.5	73.2

Soil samples of the experimental area were air-dried, passed through a two mm sieve, and examined according to the procedures described by^36^. The dominant soil type of the experimental site was loamy clay (60.7%) with pH 8.0, organic matter (3.0%), HCO-3 (0.8 meq/l), CL− (8.8 meq/l), Ca^++^ (6.1 meq/l), Mg^+^ (3.5 meq/l), and K+ (1.5 meq/l) average over the two seasons.

### Experimentation, factors, and their levels

A split-plot design with four replicates was used in both seasons. The main plot was used to investigate irrigation intervals. The irrigation intervals treatments were as follows: (1) Irrigation every 10 days (I1); (2) Irrigation every 15 days (I2) and, (3) Irrigation every 20 days (I3). The subplots were treated with K-silicate treatments as follows: (1) Spray once at 40 days after sowing (S1); (2) Spray twice at 40 and 60 days after sowing, respectively (S2) and, (3) Spray three times at 40, 60, and 80 day after sowing, respectively (S3) and, (4) Control (water spray) (S4).

The liquid K-silicate (K_2_SiO_3_; 10% K_2_O, 25% SiO_2_) was obtained from Abo Ghaneima Company Trade and Agencies, Alexandria—Abu Qir and applied at the rate of 1000 cm^3^/l. Yellow maize hybrid (SC168) was sown on June 3 and May 28 in 2017 and 2018, respectively. Maize was sown in hill spaced 25 cm, and plants were thinned to one plant per hill. Calcium superphosphate (12.5%) was added at the rate of 60 kg P_2_O_5_/ha (ha = 2.4 feddan) during soil preparation. Nitrogen fertilizer from urea (46.5% N) was added at the rate of 288 kg N/ha in two equal doses before the 1st and second irrigations.

Each sub plot consisted of 5 ridges 6 m in length and 70 cm in the width and plot area was 21 (square meter = m^2^) and the treatments were distributed randomized in 4 replicates. All the agricultural practice was done according the recommendation of Ministry of Agriculture and Land Reclamation.

### Data recording

During current study, at harvest time, the ears were harvested from the two middle ridges of each sub plot to determine the following characters i.e., the plant height (cm), ear length & diameter (cm), grain weight/ear (g), shelling percentage (%), 100-grain weight (g), biological, grain and straw yield (t/ha) were estimated. The shelling % for each treatment was determined using the following formula^37^:

$$ Shelling\;percentage = \frac{Grain\;weight\;of\;ears}{{Total\;weight\;of\;ears}} \times 100 $$

WUE was calculated according the method described by Israelsen and Hansen^37^ as follows:

$$ WUE = \frac{{Grain\;yield\;({\text{kg/area}})}}{{Total\;water\;used\;({\text{m}}^{3} {\text{/area}})}} \times 100 $$

Grain protein percentage (%) was determined according to the improved Kjeldahl method, where approximately 1 g of grains of maize was hydrolyzed with 15 mL concentrated sulfuric acid (H_2_SO_4_) containing two copper catalyst tablets in a heat block at 420 °C for 2 h. After cooling, H_2_O was added to the hydrolysates before neutralization and titration. The amount of total nitrogen in the grains were multiplied with the traditional conversion factor of 6.25^38^ in order to determine total protein content.

Grain oil percentage (%) was determined by extraction using a Soxhlet apparatus according to the methods described by the Association of Official Agricultural Chemists^39^, where maize grains were crushed and ground into a fine powder. The oil was extracted from each seed sample (30.0 g) with n-hexane (250 mL) using a Soxhlet extractor at 70 °C for 6 h. After extraction, the hexane-oil mixture was passed through a layer of anhydrous magnesium sulfate placed over filter paper in a funnel. The solvent was evaporated under vacuum using a rotary evaporator at 40 °C. The resultant oil was weighed and flushed with nitrogen and stored at − 20 °C until further analysis.

### Statistical analysis

We subjected all the parameters to analysis of variance and further statistically analyzed according to^40^ for split-plot arrangement. Also, the means between different treatments were compared using (LSD) a least significant differences test (p ≤ 0.05) in the CoStat software package^41^.

### Human and animal rights

This article does not contain any studies with human or animal subjects.

## Results

### Maize response to irrigation intervals (I)

Table 2 shows the significant effect of irrigation intervals on plant height (cm), ear length (cm), grain weight (g)/ear, and shelling percentage (%), but not ear diameter (cm), of maize during the 2017 and 2018 seasons. The tallest plant height was recorded with irrigation every 10 days in both seasons, whereas the longest ear length was obtained with irrigation each 10 and 15 days in first and second seasons, respectively. The heaviest grain weight was achieved with irrigation every 15 days in both seasons. Irrigation every 10 and 15 days recorded the highest values of shelling percentage in the first and second seasons, respectively. The lowest growth responses occurred with irrigation each 20 days in the two experiment years.

**Table 2:** Plant height, ear length, ear diameter, grain weight/ear, and shelling percentage of maize as affected by irrigation intervals and foliar application of K-silicates in the 2017 and 2018 seasons.

Characters	Irrigation intervals (I)	Season 2017								Season 2018							
		Foliar application of K-silicate (K_2_SiO_3_)					L.S.D._0.05_			Foliar application of K-silicate (K_2_SiO_3_)					L.S.D._0.05_		
		S1 (once)	S2 (twice)	S3 (three times)	S4 (control)	Average (I)	I	S	I × S	S1 (once)	S2 (twice)	S3 (three times)	S4 (control)	Average (I)	I	S	I × S
Plant height (cm)	I1 (10 days)	251.7	245.7	254.7	251.3	250.8				236.0	220.0	220.0	231.0	226.8			
	I2 (15 days)	227.0	239.3	248.3	226.7	235.3	6.7	5.4	9.3	219.0	211.3	224.7	208.7	215.9	6.9	5.0	10.3
	I3 (20 days)	229.5	238.3	251.3	223.7	235.7				200.0	198.7	205.3	194	199.5			
	Average	236.1	241.1	251.4	233.9					218.3	210.0	216.7	211.2				
Ear length (cm)	I1 (10 days)	18.53	19.07	20.4	18.8	19.2				17.2	18.3	18.6	18.8	18.2			
	I2 (15 days)	18.2	18.27	20.3	18.4	18.8	0.4	1.0	1.1	18.8	19.3	19.5	18.9	19.1	0.6	0.9	1.2
	I3 (20 days)	17.33	17.9	18.9	17.5	17.9				18.2	18.5	19.67	17.4	18.4			
	Average	18.0	18.4	19.9	18.2					18.1	18.7	19.3	18.4				
Ear diameter (cm)	I1 (10 days)	4.3	4.5	4.6	4.3	4.4				4.3	4.4	4.5	4.5	4.4			
	I2 (15 days)	4.2	4.5	4.6	4.4	4.4	ns	0.3	ns	4.3	4.4	4.5	4.2	4.3	ns	ns	ns
	I3 (20 days)	4.2	4.3	4.5	4.0	4.3				4.3	4.5	4.5	4.3	4.4			
	Average	4.2	4.4	4.6	4.2					4.3	4.4	4.5	4.4				
Grain weight/ear (g)	I1 (10 days)	149.9	171.2	171.9	154.2	161.8				150.5	168.7	177.1	151.5	162.0			
	I2 (15 days)	157.7	170.1	177.1	154.0	164.7	15.0	13.0	19.8	158.8	169.1	176.7	155.8	165.1	6.9	6.5	12.5
	I3 (20 days)	130.8	153.8	167.3	133.3	146.3				134.0	153.8	166.5	136.8	147.8			
	Average	146.1	165.0	172.1	147.2					147.8	163.9	173.4	148.0				
Shelling percentage (%)	I1 (10 days)	77.0	75.0	80.0	76.0	77.0				83.0	89.0	90.0	81.0	85.8			
	I2 (15 days)	74.0	78.0	81.0	74.0	76.8	1.5	3.5	4.3	86.0	87.0	91.0	82.0	86.5	2.6	4.1	5.8
	I3 (20 days)	70.0	72.0	75.0	69.0	71.5				77.0	81.0	85.0	73.0	79.0			
	Average	73.7	75.0	78.7	73.0					82.0	85.7	88.7	78.7				

I1, I2, and I3: irrigation intervals treatments; S1,S2, S3 and S4: foliar application of K-silicate treatments; ns, not significant; L.S.D.at 5%, the least significant difference at a 5% level of significance.

Results given in Table 3 indicate that irrigation intervals significantly affected 100-grain weight, biological yield (t/ha), grain yield (t/ha), straw yield (t/ha), and WUE in the two studied seasons. The highest 100-grain weight values were recorded with irrigation every 10 days. However, there was no significant difference among irrigation each 15 days, which gave the highest mean values of biological yield, grain yield, and straw yield per ha. Irrigation every 20 days resulted in the lowest values of these traits but achieved the highest WUE. Irrigation each 10-day interval recorded the lowest WUE values in both seasons.

**Table 3:** Yield and yield components of maize as affected by irrigation intervals and foliar application of K-silicates in the 2017 and 2018 seasons.

Treatments		2017								2018							
Characters	Irrigation intervals (I)	Foliar application of K-silicate (K_2_SiO_3_)					L.S.D._0.05_			Foliar application of K-silicate (K_2_SiO_3_)					L.S.D._0.05_		
		S1 (once)	S2 (twice)	S3 (three times)	S4 (control)	Average (I)	I	S	I × S	S1 (once)	S2 (twice)	S3 (three times)	S4 (control)	Average (I)	I	S	I × S
100-grain weight (g)	I1 (10 days)	26.6	28.2	30.6	26.5	28.0				26.9	28.6	30.1	27.2	28.2			
	I2 (15 days)	27.1	28.1	29.7	25.4	27.6	1.3	1.6	2.0	26.1	28.2	29.8	25.9	27.5	1.3	1.5	2.5
	I3 (20 days)	21.9	25.0	27.9	21.3	24.0				22.3	24.3	27.1	20.5	23.5			
	Average	25.2	27.1	29.4	24.4					25.1	27.0	29.0	24.5				
Biological yield (t/ha)	I1 (10 days)	20.4	22.1	23.3	19.7	21.4				21.8	22.6	23.5	21.6	22.4			
	I2 (15 days)	22.6	23.5	25.2	20.6	23.0	0.7	1.2	1.7	22.3	22.8	23.8	21.8	22.7	0.2	0.7	1.4
	I3 (20 days)	19.4	20.4	22.1	19.0	20.2				19.4	21.1	22.3	18.7	20.4			
	Average	20.8	22.0	23.5	19.8					21.2	22.2	23.2	20.7				
Straw yield (t/ha)	I1 (10 days)	12.2	13.4	14.2	11.8	12.9				13.4	13.9	14.2	13.2	13.7			
	I2 (15 days)	13.9	14.6	15.6	12.7	14.2	1.2	1.3	2.1	13.9	14.2	14.4	14.4	14.2	0.5	0.7	1.4
	I3 (20 days)	12.2	12.7	13.4	10.8	12.3				12.0	13.2	13.7	12.0	12.7			
	Average	12.8	13.6	14.4	11.8					13.1	13.8	14.1	13.1				
Grain yield (t/ha)	I1 (10 days)	8.2	8.6	9.1	7.9	8.5				8.4	8.6	9.4	8.4	8.7			
	I2 (15 days)	8.6	8.9	9.6	7.9	8.8	0.6	0.1	0.7	8.4	8.6	9.4	7.4	8.5	0.7	0.6	0.9
	I3 (20 days)	7.2	7.7	8.6	8.2	7.9				7.4	7.9	8.6	6.7	7.7			
	Average	8.0	8.4	9.1	8.0					8.1	8.4	9.1	7.5				
Water use efficiency (WUE) (kg/m^3^)	I1 (10 days)	1.1	1.2	1.3	1.0	1.2				1.1	1.1	1.2	1.0	1.1			
	I2 (15 days)	1.5	1.6	1.8	1.5	1.6	0.2	0.1	0.2	1.4	1.4	1.8	1.3	1.5	0.1	0.1	0.1
	I3 (20 days)	1.8	1.8	2.1	1.6	1.8				1.4	1.6	1.9	1.5	1.6			
	Average	1.5	1.5	1.7	1.4					1.3	1.4	1.6	1.3				

I1, I2, and I3: irrigation intervals treatments; S1,S2, S3 and S4: foliar application of K-silicate treatments; L.S.D.at 5%, the least significant difference at a 5% level of significance.

The irrigation every 10 days increased 100-grain weight by 14.29 and 16.67% as compared with interval irrigation 20 days in the first and the second seasons, respectively. While irrigation every 15 days increased biological yield by 12.17 and 10.13%, straw yield by 13.38 and 11.97%, as compared with interval irrigation 20 days in the first and the second seasons, respectively. On the other hand, grain yield increased by 10.23 and 11.49% with irrigation every 15 and 10 days as compared with interval irrigation 20 days in the first and the second seasons, respectively.

Table 4 shows the effect of irrigation intervals on grain protein and oil content, where irrigated maize plants every 15 days (I2) recorded the highest mean values of grain protein content (%), on the other hand, irrigation interval (10 days) gave the highest value of oil content in grain in the two seasons.

**Table 4:** Grain protein content (%) and content oil (%) of maize as affected by irrigation intervals and foliar application of K-silicates in the 2017 and 2018 seasons.

Treatments		2017								2018							
Characters	Irrigation intervals (I)	Foliar application of K-silicate (K_2_SiO_3_)					L.S.D._0.05_			Foliar application of K-silicate (K_2_SiO_3_)					L.S.D._0.05_		
		S1 (once)	S2 (twice)	S3 (three times)	S4 (control)	Average (I)	I	S	I × S	S1 (once)	S2 (twice)	S3 (three times)	S4 (control)	Average (I)	I	S	I × S
Grain protein content (%)	I1 (10 days)	9.2	9.7	9.9	8.9	9.4				9.4	9.8	10.0	9.0	9.5			
	I2 (15 days)	9.6	9.8	11.0	10.0	10.1	0.5	0.7	1.1	10.1	10.5	11.2	9.2	10.2	0.4	0.6	1.0
	I3 (20 days)	9.7	10.2	10.4	8.9	9.8				10.0	10.1	10.1	8.8	9.8			
	Average	9.5	9.9	10.5	9.3					9.8	10.1	10.4	9.0				
Grain oil content (%)	I1 (10 days)	3.6	3.6	3.9	3.3	3.6				3.6	3.6	3.9	3.4	3.6			
	I2 (15 days)	3.4	3.5	3.7	3.4	3.5	0.3	0.4	0.5	3.4	3.6	3.8	3.5	3.6	0.3	0.3	0.6
	I3 (20 days)	3.3	3.3	3.2	3.2	3.3				3.3	3.2	3.3	3.3	3.3			
	Average	3.4	3.5	3.6	3.3					3.5	3.5	3.7	3.4				

I1, I2, and I3: irrigation intervals treatments; S1,S2, S3 and S4: foliar application of K-silicate treatments; L.S.D.at 5%, the least significant difference at a 5% level of significance.

### Maize response to K-silicate (S)

Table 2 shows the significant effect of foliar usage of K-silicate on plant height, ear length, ear diameter, grain weight, and shelling percentage of maize during the 2017 and 2018 seasons. The tallest plant height, longest ear length, largest ear diameter, and heaviest grain weight were recorded with three foliar applications of K-silicate. The lowest yield parameters were recorded with water spray (control = S4) treatments in both seasons.

Results in Table 3 revealed that K-silicate had significant effects on 100-grain weight, biological yield/ha, grain yield/ha, straw yield/ha, and WUE in both seasons. As compared with other treatments, the highest values occurred when maize plants were sprayed K-silicate three times (S3), whereas the lowest values occurred with foliar water spray (control = S4) treatments in 2017 and 2018 seasons. 100-grain weight increased with increasing K-silicate spraying three times by 17.01 and 15.52%, biological yield increased by 15.74 and 10.78%, straw yield increased by 18.06 and 7.09%, grain yield increased by 12.09 and 17.58% and water use efficiency (WUE) increased by 17.65 and 18.75 as compared with the control treatments (spray water) in the first and the second seasons, respectively.

Table 4 shows significant differences between foliar spray treatment and control on grain protein and oil contents in both seasons. Foliar spraying with K-silicate three times (S3) recorded the highest values of these measures.

### Maize response to interaction between irrigation intervals and K-silicate (I × S)

The interaction results among irrigation intervals and foliar spray with K-silicate had major effect on plant height, ear length, grain weight, and shelling percentage, but not ear diameter, in both the 2017 and 2018 seasons (Table 2). The greatest values of these traits were recorded when maize plants were irrigated every 15 days (I2) under foliar spraying with K-silicate three times (S3), whereas the lowest values resulted from irrigation every 10 or 20 days under water spray (control) in both cropping seasons.

The interaction values between irrigation intervals and foliar spray with K-silicate had considerable effect on 100-grain weight, biological yield, grain yield, straw yield, and WUE in both seasons (Table 3). The maximum values of these traits were recorded when maize plants were irrigated every 15 days (I2) under foliar spraying with K-silicate three times (S3) in both seasons, whereas the lowest ones were given with irrigation every 10 or 20 days under water spray (control) in both cropping seasons. WUE provides information on the adaptation potential of a plant to water stress conditions, making it an important characteristic. WUE under irrigation every 20 days (I3) interval and foliar spraying with K-silicate three times (S3) gave the highest values, followed by irrigation interval (I2), under the same foliar spray (S3) in 2017 and 2018 seasons. Foliar spraying of water (S4) recorded the lowest values under irrigation each 10 days (I1) in both seasons (see Table 3). The interaction of irrigation and Si treatment had a significant effect on protein content in both seasons. Treatment S3 recorded the highest values, whereas irrigation every 15 days (I3) and water spray control treatments (S4) gave the lowest values in both seasons. The interaction had significant effect on grain oil content. Foliar spraying with K-silicate three times (S3) recorded the highest values. Irrigation every 15 days (I3) combined with water spray control treatments (S4) gave the lowest values in both seasons (Table 4).

## Discussion

These results support those obtained by^42^, who reported that water stress significantly decreased growth, grain yield, and water characteristics as WUE. Also^29^, found that increasing the irrigation interval from 8 to 14 and 20 days caused a significant decrease in growth and yield of the crop. Shi et al.^43^ showed WUE was higher in the dry-cultivation treatment since yields decreased relatively less than the supply of irrigation water. However, higher WUE can be achieved by relating deficit stress at the late vegetative stage somewhat than maturation stage^44^.

The reduction of yield, yield component and quality under waters stress could be due to numerous reasons including decrease of photosynthesis efficiency, leaf area, net assimilation production, and reduction of water and mineral absorption by the root which ultimately decline developmental and vegetative growth^11^**.**

The results indicate that increasing irrigation intervals to 20 day reduced all the studied characteristics likely due to water stress deficit. Inadequate available soil water reduces the metabolic activity of maize, decreases its dry matter accumulation, and reduces its photosynthetic level by reducing the chlorophyll content in leaves. These changes eventually lead to a loss in maize yield^45^. Further, water stress had a greater effect on the growth and development of maize through the seedling stage than the other three stages. Water stress reduced growth and biomass due to decreased intercepted photosynthetically active radiation and radiation-use efficiency. These effects extended into the reproductive stage and finally decreased grain weight and yield^45^. Water deficit stress through the later vegetative and maturation stage directly decreased the grain-filling level and period and thus had the greatest effect on grain yield and its components. Yield was reduced after water-deficit stress occurred during the late vegetative stage and was exacerbated by additional stress during the maturation stage. In all treatments, yield decrease was proportional to the severity of the water stress. However, water stress used had a larger effect on maize yield during the maturation stage than during the late vegetative stage^46^.

These findings support previous investigations by^47^ who showed that weekly K-silicate foliar spraying resulted in a significant effect on growth and yield parameters of maize. Furthermore, using K-silicate gave high values of crop growth, grain yield, and quality^48^. In this study, foliar applications of K-silicate at 40, 60, and 80 days after sowing recorded the highest values of all the studied characteristics under the regional conditions. These values may be due to the role of K-silicate in increasing the utilization rate and nutrient absorption^49^. Foliar application of K-silicate has also been shown to enhance leaf erectness, increase photosynthesis efficiency, and minimize lodging capability in grasses^50^. Si application increased growth and yield, improved strength, lessened climate stress, supplied impedance to mineral stress^51^, and increased WUE compared to the control^52^.

The increase of growth, yield, quality, and WUE under water stress may be due to the vital role of K-silicate in reducing water-deficit stress on plant growth and yield. These results corroborate those of^53^ who reported that the sorghum yield increased by irrigation with 14-day interval and spraying K-silicate. Thus, Si can mediate drought tolerance^51^. Moreover^24^, observed that the three are increase in growth, yield, protein by foliar application of K-silicate under water stress. Spraying K-silicate has the potential to alleviate the negative effects of drought stress on yield and quality of sugar beet^29^. Si foliar application has bio-stimulative impacts because it alleviates stressful conditions such as salinity, deficiency or excess of water, high and low temperature, and the stout pressure of diseases and pests. Si application to plants is unusual. However, Si fertilization can be effective through foliar application, which is much cheaper and more convenient to use than soil fertilization^54^.Also, foliar application of Si moderated the unfavorable impacts of environmental stresses on the yield of many plants^55^. Research has shown that Se played a critical role in virous plants, eventually afecting crop yield, factors such as starch accumulation in chloroplast, resistance improvement to oxidative stress, postponing of senescence, and water status adjustment under stress conditions and enhnace of antioxidative capacity^56^.

## Conclusions

Form the above obtained results, the addition of K-silicate at the three times through foliar application (40, 60, and 80 days after sowing) at rates 1000 cm^3^/l is beneficial to alleviate the adverse effects of water-deficit stress. The possible mechanism involved in the improvement of growth, yield, and quality characters is the increase of WUE in plants. K supply within this range significantly improved the macronutrient uptake of maize plants. Based on this investigation, under water shortage, we can recommend that the application of K-silicate three time through foliar spraying, which can increase WUE and can result in high grain yield, its components and quality such grain protein content and oil content (%) under irrigation every 15-day interval under the study conditions and the similar conditions.
